# Evidence for spinal disinhibition as a pain-generating mechanism in fibromyalgia syndrome

**DOI:** 10.1097/PR9.0000000000001236

**Published:** 2024-12-26

**Authors:** Anne Marshall, Jamie Burgess, Andreas Goebel, Bernhard Frank, Uazman Alam, Andrew Marshall

**Affiliations:** a Pain Research Institute, Institute of Life Course and Medical Sciences, University of Liverpool, Liverpool, United Kingdom; b University Hospital Aintree, Liverpool University Hospitals NHS Foundation Trust, Liverpool, United Kingdom; c Department of Pain Medicine, The Walton Centre NHS Foundation Trust, Liverpool, United Kingdom; d Diabetes and Endocrinology Research, Liverpool Centre for Cardiovascular Science, Institute of Life Course and Medical Sciences University of Liverpool, Liverpool, United Kingdom; e Visiting Fellow Centre for Biomechanics and Rehabilitation Technologies Staffordshire University, Stoke-on-Trent, United Kingdom; f Department of Clinical Neurophysiology, The Walton Centre NHS Foundation Trust, Liverpool, United Kingdom

**Keywords:** Fibromyalgia, Spinal disinhibition, H-reflex rate-dependent depression, Conditioned pain modulation

## Abstract

**Introduction:**

Pain phenomenology in patients with fibromyalgia syndrome (FMS) shows considerable overlap with neuropathic pain. Altered neural processing leading to symptoms of neuropathic pain can occur at the level of the spinal cord, and 1 potential mechanism is spinal disinhibition. A biomarker of spinal disinhibition is impaired H-reflex rate-dependent depression (HRDD).

**Objectives:**

This study investigated whether patients with FMS exhibit evidence of spinal disinhibition.

**Methods:**

Thirty-one individuals with FMS and 20 healthy volunteers underwent testing of Hoffman reflex including HRDD, along with assessment of clinical signs and symptoms, pressure pain thresholds, temporal summation of pain (wind-up), and conditioned pain modulation (CPM). Small nerve fibre structure was quantified using intraepidermal nerve fibre density and corneal confocal microscopy.

**Results:**

Patients with FMS had significantly impaired HRDD at 1 Hz (*P* = 0.026) and 3 Hz (*P* = 0.011) and greater wind-up ratio (*P* = 0.008) compared with healthy controls. Patients with the most impaired HRDD also had the most inefficient CPM but HRDD was not associated with wind-up. Both HRDD and CPM were most impaired in patients with a shorter duration of disease.

**Conclusion:**

We demonstrate for the first time that people with FMS show evidence of spinal disinhibition, which is most dominant in shorter duration of disease and may represent a putative mechanism of pain generation in FMS. Identifying people with impairment of central pain processing at an early stage may provide opportunities for targeted mechanistically directed interventions. Longitudinal studies are warranted to tease out the precise contribution of these mechanisms.

## Abstract 2

Supplemental Digital Content is Available in the Text.

Individuals with fibromyalgia syndrome have impaired Hoffman reflex rate-dependent depression implicating spinal disinhibition as a pain-generating mechanism and novel treatment target.

## 1. Introduction

Fibromyalgia syndrome (FMS) is a common but poorly understood condition. It is a frequent cause of chronic widespread pain, often accompanied by fatigue, cognitive impairment, sleep, and mood disturbances.^7^ This complex and heterogenous syndrome presents challenges to both diagnosis and treatment,^6^ partly due to a lack of understanding of its pathoaetiology. It is debated whether the symptoms and signs of FMS reflect primarily peripheral or central sensitisation.^8,10,15,40,44,65^ People with FMS frequently report symptoms and signs seen in neuropathic pain including paraesthesia, hyperalgesia, and allodynia. Indeed, a subset of patients with FMS develops small fibre pathology,^15,25,63^ and microneurography studies in FMS demonstrate c-fibre nociceptor sensitisation.^54^

Whilst in some patients with pain syndromes, the underlying pathophysiology relates to peripheral nerve fibre degeneration and sensitisation, the functional significance of small fibre abnormalities and their relation to symptoms in FMS remains unclear.^17,35,62^ Furthermore, it is recognised that in FMS changes in the brain^2,16,51^ and spinal cord^56,68^ may generate, maintain, and modulate pain signalling. Both reduced pain modulation with inefficient descending pain inhibition,^36,69^ and/or increased temporal summation of pain,^55^ are reported in patients with FMS. However, recent studies in FMS using sensitivity-adjusted stimuli demonstrate effective processing of nociceptive input.^55–58^ Another potential mechanism of central sensitisation is spinal disinhibition, where disruption of intrinsic ligand-gated ionotropic inhibitory systems in the dorsal horn of the spinal cord leads to the amplification of incoming peripheral signals and increased ascending nociceptive drive.^50^ Spinal disinhibition has been extensively studied in preclinical neuropathic and inflammatory pain models^50^ and is proposed as a novel treatment target. However, the lack of a biomarker of spinal disinhibition as a pain-generating mechanism in humans has hindered the investigation of spinal inhibitory dysfunction in clinical pain conditions.

Noninvasive investigation of human spinal circuits using neurophysiological methods, for example, the Hoffman reflex (H-reflex), can provide valuable information about excitation and inhibition in the spinal cord.^43^ Hoffman reflex testing elicits a direct M wave and a longer latency trans-spinal H wave. The H-wave response is classically considered a monosynaptic circuit between type 1a muscle spindle afferents and motoneurons in the ventral spinal cord. However, it is subject to modification by intrinsic inhibitory oligo- and polysynaptic spinal circuits as well as supraspinal influences.^4,34^ These influences modulate the H-wave amplitude by altering the balance between excitatory and inhibitory inputs in the H-reflex pathway. An example, the diminution of the amplitude of the H wave with consecutive stimulations is termed H-reflex rate-dependent depression (HRDD).^28^

Translational studies indicate that impaired HRDD is a biomarker of spinal disinhibition, likely due to chloride dysregulation in the dorsal horn of the spinal cord, in animals and humans.^33,34^ We have previously demonstrated that HRDD is impaired in patients with painful diabetic neuropathy.^38,68^ Whether patients with FMS have evidence of spinal disinhibition is unknown. The primary aim of this study was to determine, using HRDD, whether patients with FMS display evidence of spinal disinhibition. We also aimed to determine whether HRDD is associated with presumed changes in central processing, wind-up, and conditioned pain modulation (CPM), as well as primary psychophysical characteristics of FMS.

## 2. Methods

Thirty-one patients with FMS were recruited to the DEFINE-FMS study (South West—Frenchay Research Ethics Committee—20/SW/0138) from physiotherapy-led musculoskeletal fibromyalgia services, pain clinics, as well as community-based fibromyalgia patient support groups. Patients underwent testing of HRDD, assessment of pressure pain thresholds (PPTs), temporal summation of pain and CPM, corneal confocal microscopy, quantification of intraepidermal nerve fibre density (IENFD) using skin biopsy, and completion of questionnaires to assess the presence of pain, neuropathic symptoms, and the impact of FMS on day-to-day life. To be included in this study, people with FMS aged ≥18 years satisfied the modified American College of Rheumatology 2016 diagnostic criteria.^66^ Twenty healthy volunteers (HV) were also recruited.

### 2.1. Assessment of H-reflex rate-dependent depression

**Figure 1.**
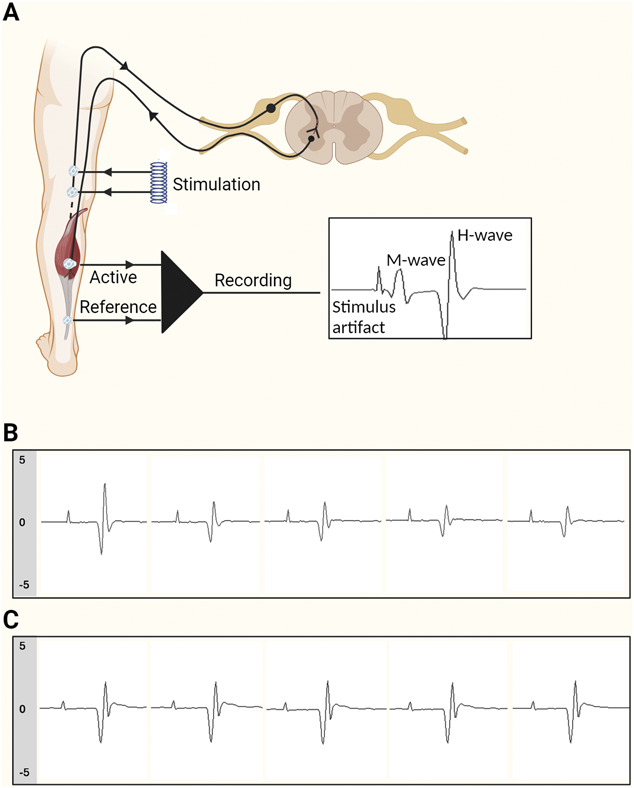
The H-reflex and HRDD. (A) Schematic representation for eliciting and recording the H-reflex (created with Biorender). (B) Representative trace of HRDD, using 1-Hz stimulation, in an individual with normal HRDD. (C) Representative trace of HRDD, using 1-Hz stimulation, in an individual with impaired HRDD. H-reflex, Hoffman reflex; HRDD, H-reflex rate-dependent depression.

For H-reflex studies, tibial nerve stimulation was performed using 1-ms square wave monophasic pulses delivered using surface silver–silver chloride electrodes, to the popliteal fossa. Surface silver–silver chloride recording electrodes with a diameter of 9 mm were placed on the long axis of the soleus muscle (Fig. 1A). Hoffman reflex recruitment curves were obtained to determine peak–peak H-wave and M-wave maximal amplitude, by incrementing stimulation current by 1 mA (1-ms duration). A random interstimulation interval with a minimum of 10 seconds was observed. For HRDD, a submaximal stimulus strength (to achieve a response of 75% of maximum H-reflex on the rising phase of the recruitment curve) was used. H-wave responses were recorded by assessing trains of 10 stimuli delivered at 1 and 3 Hz, to demonstrate that rate dependency is preserved in individuals with FMS. Traces were inspected to ensure they were free of volitional electromyographic activity. Hoffman reflex rate-dependent depression was calculated as the mean H-reflex amplitude of responses 2 to 5 of a stimulus train, expressed as a percentage of the amplitude of the first recorded H-reflex in the train^67^ (Fig. 1B, C). Therefore, a higher value of HRDD indicates a smaller degree of depression, which we will refer to as impairment of HRDD. Pain scores (NRS 0–10) were collected following each stimulus train.

### 2.2. Questionnaires

Participants completed 5 questionnaires to assess the presence of pain, neuropathic symptoms, and the impact of FMS on day-to-day life. The Revised Fibromyalgia Impact Questionnaire was administered to determine the severity of participants’ symptoms and functional impairment, including physical impairment, ability to work, restfulness, and mood.^1^ The Neuropathy Symptom Profile was used to assess sensory, autonomic, and motor neuropathy symptoms.^14^ The small Fibre Neuropathy Screenings List evaluated symptoms of purely small nerve fibre-related neuropathy.^27^ The PainDETECT screening tool evaluated neuropathic pain symptoms.^18^ An additional measure of current pain score was assessed using the McGill visual analogue scale (0–10).^42^

### 2.3. Corneal confocal microscopy

Participants underwent corneal examination with the Heidelberg Retina Tomograph 3 with Rostock Cornea Module (Heidelberg Eye Explorer; Heidelberg Engineering GmBH, Heidelberg, Germany), and images of the corneal subbasal nerve plexus were captured following an established protocol.^59^ Image selection was masked to the FMS/control group, and the average number of images for analysis per participant was 16. Automated analysis was conducted using ACCMetrics software (ACCMetrics: Malik Lab, Imaging Science, University of Manchester). Three corneal nerve parameters were quantified from each image: (1) corneal nerve fibre density (total number of main nerve fibres per square millimetre of corneal tissue [fibre no/mm^2^]); (2) corneal nerve branch density (number of branches of all main nerve fibres per square millimetre of corneal tissue [branch no./mm^2^]); and (3) corneal nerve fibre length (the total length of all main nerve fibres and branches [mm/mm^2^] within the images).^59^

### 2.4. Skin biopsy

Participants underwent punch biopsies to assess IENFD of the lateral proximal and distal thigh and distal leg, in accordance with a previously published protocol.^3^

### 2.5. Pressure pain threshold

Pressure pain threshold was evaluated using a pressure algometer (FDN200; Wagner Instruments, Riverside, CT) with a blunt contact area of 1 cm^2^ placed on the thenar eminence. The threshold was determined as the arithmetic mean of 3 recordings, and the raw data were log transformed and converted into a *z*-score to normalize the data for age, sex, and body site tested. Positive *z*-score values denote a gain in function and negative *z*-scores denote a loss of function.^52^ Values less than −1.96 (loss of function) or greater than 1.96 (gain of function) are considered abnormal.

### 2.6. Temporal summation of pain—wind-up

Wind-up ratio was assessed using a 256-mN pinprick stimulator. If the participant found testing too painful, a 128-mN pinprick stimulator was used. A train of 10 stimuli was applied to the dorsum of the right arm at a frequency of 1 per second. A pain score, using a 0 to 100 numerical rating scale, was recorded for the initial stimulus and the subsequent train of stimuli. The wind-up ratio was calculated as the arithmetic mean of the pain intensity rating for the series of stimuli divided by the arithmetic mean of the pain intensity rating for the single stimulus. The raw data were log transformed and converted into a *z*-score to normalize the data for age, sex, and body site tested. Positive *z*-score values denote a gain in function, and negative *z*-scores denote a loss of function.^52^ Values less than −1.96 (loss of function) or greater than 1.96 (gain of function) are considered abnormal.

### 2.7. Conditioned pain modulation

Conditioned pain modulation was used to assess the efficiency of diffuse noxious inhibitory control. The pressure pain threshold on the right abductor pollicis brevis was used as the test stimulus. A pressure algometer (FDN200; Wagner Instruments) with a blunt contact area of 1 cm^2^ was placed on the skin above the abductor hallucis muscle on the right hand. Pressure was applied with increasing intensity at a rate of 0.5 kg (50 kPa)/s. The participant indicated the moment the sensation of pressure changed to an additional painful “burning,” “stinging,” or “aching” sensation, and the respective value on the algometer was recorded. The test was repeated 3 times with a break of 10 seconds in between, and the mean value was recorded. The cold-pressor test was used as the conditioning stimulus, with the left hand of the patient immersed up to the wrist in a water bath of melting ice water for up to 180 seconds or as long as the participant could tolerate, with a minimum time of 45 seconds. Pain ratings, using a numerical rating scale of 0 to 100, were recorded every 15 seconds. Following the removal of the hand from the water bath, the test stimuli were repeated on the right hand (non-submerged) as detailed above. The CPM effect was calculated as the difference (preconditioning stimulus minus post) in raw PPTs. A negative value indicates efficient CPM.

### 2.8. Statistical methods

Statistical analyses were performed using GraphPad Prism statistical software (GraphPad Software, Inc, La Jolla, CA) and SPSS Version 29 (IBM). Continuous data (including HRDD, CPM, PPT, wind-up ratio, IENFD, and corneal confocal microscopy (CCM) parameters) were assessed for normality using quantile–quantile plots and Shapiro–Wilk test. Normal distributed data were reported as mean ± SD and analysed using an unpaired *t* test (FMS/HV) for between-group comparison. For nonnormally distributed data, the results were reported as median ± interquartile range (IQR) and analysed using Mann–Whitney test (FMS/HV) and Kruskal–Wallis test followed by a Dunn test (short-duration FMS/long-duration FMS/HV) for between-group comparison. Significance (*P*) was reported for values reaching the significance threshold set at <0.05. Correlations were performed using the Spearman rank test and expressed as a coefficient (*r*) with *P* values. Categorical data were analysed using χ^2^ Fisher exact test of association.

## 3. Results

Table 1 details the demographic, biochemical, clinical, and neuropathic characteristics of the study cohort. Data from 31 individuals with FMS and 20 healthy volunteers were included in the analysis. The details of current pain medication are documented in Supplementary Table 1 (http://links.lww.com/PR9/A279). Systematic analysis of medication was not completed due to the large degree of variability.

**Table 1:** Demographic, biochemical, H-reflex rate-dependent depression, small nerve fibre parameters, and clinical characteristics in patients with fibromyalgia syndrome and healthy volunteers.

	FMS (n = 31)	HV (n = 20)	*P*
Sex (F/M)	29/2	15/5	0.060
Age (y)	49 (35–57)	43 (31–58)	0.105
Duration of diagnosis (y)	7 (3–10)	—	—
Duration of symptoms before diagnosis	4 (2–8)	—	—
BMI (kg/m^2^)	29.3 (22.9–34.3)	24.7 (23.4–73.0)	0.198
BP (systolic) (mm Hg)	129 ± 18	126 ± 12	0.760
BP (diastolic) (mm Hg)	81 12	76 6	0.058
HbA1c (mmol/mol)	36.1 ± 5.2	31.2 ± 7.8	0.309
Cholesterol (mmol/L)	5.0 ± 1.1	4.8 ± 0.8	0.498
Triglycerides (mmol/L)	1.5 ± 0.8	1.4 ± 0.7	0.731
CNFD	26.0 ± 6.1	27.1 ± 4.7	0.400
CNBD	34.9 ± 15.7	42.6 ± 22.9	0.196
CNFL	15.2 ± 3.2	16.8 ± 5.4	0.215
IENFD proximal thigh	8.7 ± 4.2	11.3 ± 5.5	0.502
IENFD distal thigh	9.0 ± 5.1	10.7 ± 8.2	0.441
IENFD distal leg	7.6 ± 3.6	8.3 ± 5.3	0.618
Pressure pain threshold (*z*-score)	3.79 ± 2.02	0.44 ± 1.68	**<0.001**
HRDD at 1Hz	50.9 (42.6–68.7)	41.8 (34.6–57.5)	**0.026**
HRDD at 3Hz	46.8 (35.6–57.2)	32.3 (25.2–40.9)	**0.011**
Depression (Y/N)	28/3	1/19	**<0.001**
Depression (score out of 10)	6 (2–8)	0 (0–0)	**<0.001**
Anxiety (Y/N)	29/2	6/13	**<0.001**
Anxiety (score out of 10)	7.5 (4–8)	0 (0–2)	**<0.001**
FIQR total (score out of 100)	76 (50–81)	0 (0–4)	**<0.001**
SFNSL	47 (37–60)	2 (1–6)	**<0.001**
PainDetect total (out of 38)	23 (17–28)	0 (0–1)	**<0.001**
Neuropathy symptom profile	20 (16–25)	0 (0–1)	**<0.001**
VAS current pain score	80 (68–88)	0 (0–17)	**<0.001**

Parametric data are shown as mean ± SD and analysed using an unpaired *t* test. Nonparametric data are shown as median (interquartile range) and analysed using the Mann–Whitney test. Values reaching the significance threshold set at <0.05 are in bold.

BMI, body mass index; BP, blood pressure; CNFD, corneal nerve fibre density; CNBD, corneal nerve branch density; CNFL, corneal nerve fibre length; FIQR, fibromyalgia impact questionnaire (revised); HbA1c, glycated haemoglobin; HV, healthy volunteers; HRDD, Hoffmann reflex rate-dependent depression; IENFD, intraepidermal nerve fibre density; SFNSL, small fibre neuropathy screening list; VAS, visual analogue scale.

### 3.1. Demographic, biochemical, and clinical characteristics

There was no difference in age, body mass index (BMI), or glycated haemoglobin (HbA1c) between FMS and healthy volunteers. In keeping with previous studies,^19,29,46^ approximately half (48.4%) of the individuals with FMS had IENFD below the normative range for age and sex in at least 1 location and 24% had an abnormality in corneal nerve fibre morphology.^47,60^ Whilst mean IENFDs and CCM parameters in patients with FMS were reduced compared with healthy volunteers, this did not reach a level of significance. As expected, PPT (FMS: mean 3.79, SD 2.02; HV: mean 0.44, SD 1.68; *P* < 0.001) was reduced (gain of function) in patients with FMS, and scores on pain and symptom questionnaires (Revised Fibromyalgia Impact Questionnaire; Small Fibre Neuropathy Screenings List; Neuropathy Symptom Profile; PainDetect; visual analogue scale, all *P* < 0.001) were higher in people with FMS compared with healthy volunteers. Frequency and severity of anxiety (FMS: median 7.5, IQR 4–8 [n = 29/31]; HV: median 0, IQR 0–2 [n = 6/20]; *P* < 0.001) and depression (FMS: median 6, IQR 2–8 [n = 28/31]; HV: median 0, IQR 0-0 [n = 1/20]; *P* < 0.001) were higher in patients with FMS compared with healthy volunteers.

### 3.2. Conditioned pain modulation and temporal summation of pain

**Figure 2.**
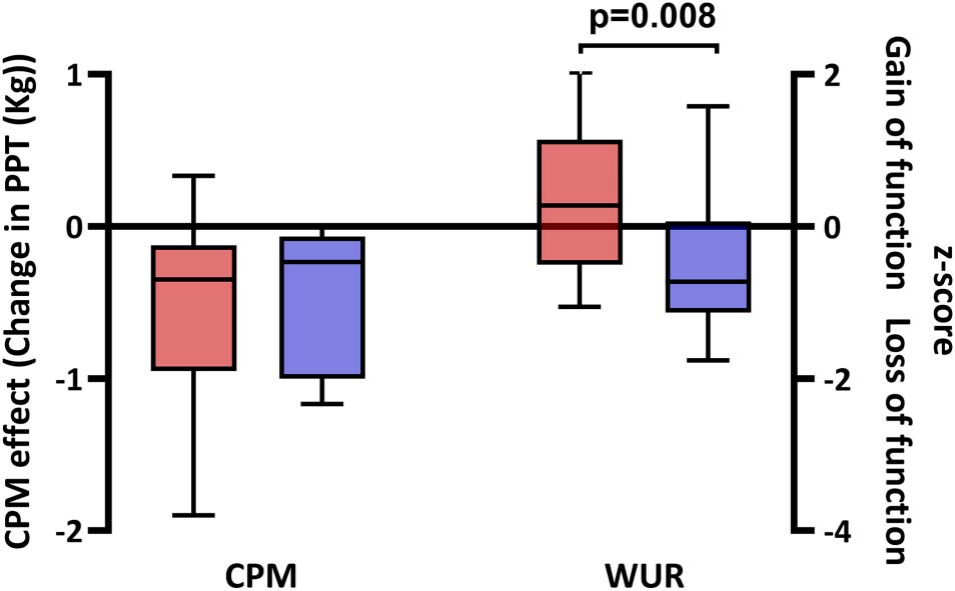
Conditioned pain modulation and temporal summation of pain in FMS. Box and whisker plot of CPM and temporal summation of pain (wind-up) in patients with FMS (red) and healthy volunteers (purple). Statistically significant *P* values are shown (Mann–Whitney). CPM, conditioned pain modulation; FMS, fibromyalgia syndrome; PPT, pressure pain threshold; WUR, wind-up ratio.

The level of CPM did not differ between people with FMS and healthy volunteers. Temporal summation of pain was increased in people with FMS compared with healthy volunteers (Fig. 2).

### 3.3. Hoffman reflex parameters

Table 2 details the H-reflex parameters in patients with FMS and healthy volunteers.

**Table 2:** Hoffman reflex parameters in patients with fibromyalgia syndrome and healthy volunteers.

	FMS	HV	*P*
Hmax	3.70 (2.17–4.60)	2.21 (1.96–4.41)	0.534
Mmax	5.78 (4.11–8.20)	7.86 (4.61–8.80)	0.509
Hmax/Mmax ratio	0.59 (0.52–0.67)	0.31 (0.11–0.62)	0.094

Data are shown as median (interquartile range) and analysed using the Mann–Whitney test. Statistically significant *P* values are shown (Mann–Whitney).

FMS, fibromyalgia syndrome; Hmax, maximum amplitude of the H wave; Hmax/Mmax ratio, ratio of the maximum amplitudes of H and M waves; HV, healthy volunteers; Mmax, maximum amplitude of the M wave.

There was no significant difference in Hmax, Mmax, or Hmax/Mmax ratio between individuals with FMS and healthy volunteers.

### 3.4. Hoffman reflex rate-dependent depression

**Figure 3.**
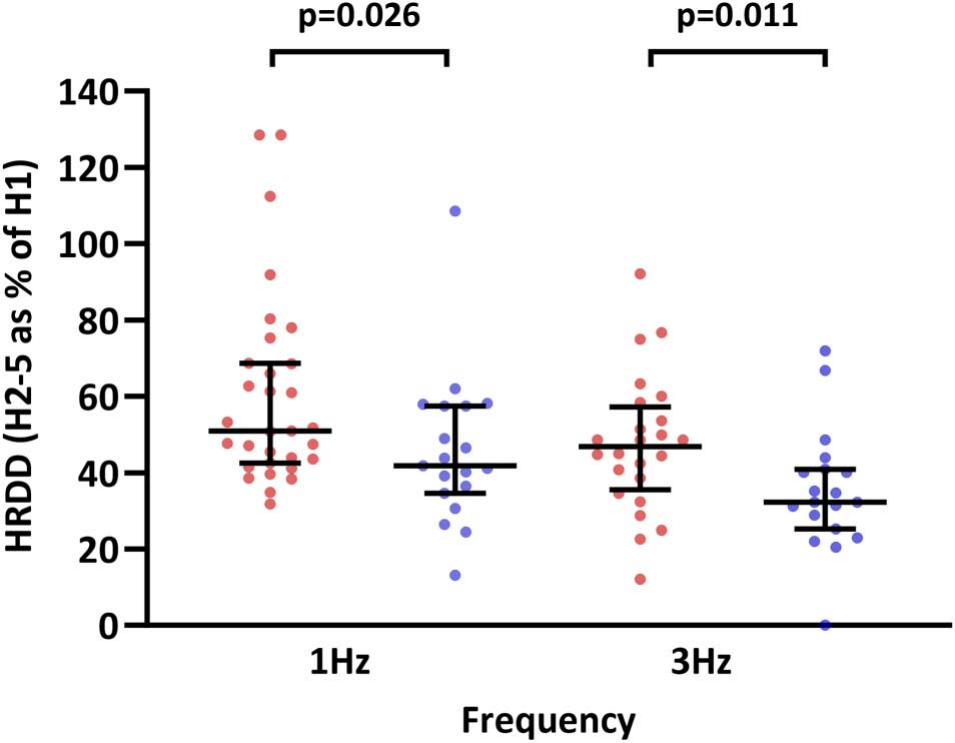
Hoffman reflex rate-dependent depression in FMS. Hoffman reflex rate-dependent depression at 1 and 3 Hz in patients with FMS (red circles) and healthy volunteers (purple circles). Statistically significant *P* values are shown (Mann–Whitney). FMS, fibromyalgia syndrome; HRDD, H-reflex rate-dependent depression.

Individuals with FMS had impaired HRDD at 1 Hz (FMS: median 50.9, IQR 42.6–68.7; HV: median 41.8, IQR 34.6–57.5; *P* = 0.026) and 3 Hz (FMS: median 46.r8, IQR 35.6–57.2; HV: median 32.3, IQR 25.2–40.9; *P* = 0.011) compared with healthy volunteers (Fig. 3). Whilst a degree of overlap is observed, 13 patients with FMS (42%) had HRDD value (s) that fell outside the normal range of the healthy volunteers (>65.8 [45.73 + 20.06] at 1 Hz, >51.3 [35.20 + 16.08] at 3 Hz). Pain scores obtained during the HRDD trains were significantly higher in patients with FMS compared with healthy volunteers (1 Hz; FMS: median 6.0, IQR 4.0–6.23; HV: median 1.0, IQR 0–4.5; *P* = <0.001. Three Hertz; FMS: median 8.0, IQR 6.5–10; HV: median 4.0, IQR 1.25–6.25; *P* = <0.01). However, these pain scores were not associated with the degree of HRDD in either individuals with FMS (1 Hz: *P* = 0.180, *r*s = 0.277; 3 Hz: *P* = 0.387, *r*s = 0.199) or healthy volunteers (1 Hz: *P* = 0.471, *r*s = 0.192; 3 Hz: *P* = 0.408, *r*s = −0.213). No significant correlations were observed between HRDD and age, BMI, HbA1c, markers of small fibre pathology on skin biopsy and corneal confocal microscopy, or any pain and symptoms scores in individuals with FMS and healthy volunteers.

### 3.5. Hoffman reflex rate-dependent depression, conditioned pain modulation, and duration of disease diagnosis

**Figure 4.**
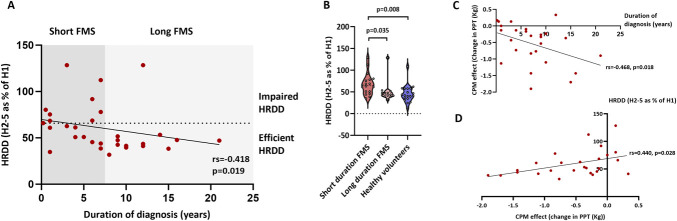
Hoffman reflex rate-dependent depression during FMS disease progression. (A) Individual H-reflex rate-dependent depression (HRDD) data in patients with FMS with short (7 years or less) and long (greater than 7 years) disease duration. Spearman correlation (*r*s) and *P* value are reported. (B) Group HRDD data in patients with FMS with short (7 years or less) and long (greater than 7 years) disease duration and healthy volunteers. Kruskal–Wallis/Dunn test *P* values are reported. (C) Conditioned pain modulation and duration of disease. Spearman correlation (*r*s) and *P* values are reported. (D) Hoffman reflex rate-dependent depression and CPM. Spearman correlation (*r*s) and *P* values are reported. CPM, conditioned pain modulation; FMS, fibromyalgia syndrome; HRDD, H-reflex rate-dependent depression.

Individuals with FMS with the most impaired HRDD also had the most inefficient CPM (*r*^s^ = 0.468, *P* = 0.018), and both HRDD (*r*^s^ = −0.418, *P* = 0.019) and CPM (*r*^s^ = −0.440, *P* = 0.028) were most impaired in patients with the shortest disease duration. To further investigate these findings, patients with FMS were divided into 2 groups based on the median duration of FMS diagnosis.^23^ Patients with a short duration (7 years or less [n = 18]) had significantly more impaired HRDD compared with patients with a long duration (>7 years [n = 13]) (Fig. 4).

## 4. Discussion

This study reports for the first time evidence of spinal disinhibition in patients with FMS. The main finding is an impairment of HRDD, a biomarker of spinal inhibitory dysfunction, in ∼40% of patients with FMS. In addition, we provide evidence that this potential mechanism of pain generation is more pronounced in shorter duration of disease.

Multiple lines of evidence in preclinical studies indicate that spinal disinhibition is a prominent mechanism for the amplification of ascending nociceptive information in the context of both neuropathic and inflammatory pain.^11,34^ The effects of this disinhibition are to reduce the threshold for activation of ascending nociceptive pathways to peripheral stimulation.^32^ Whilst spinal disinhibition could potentially arise from several convergent pathways, a dominant mechanism appears to be the disruption of the chloride balance of postsynaptic neurons so that gamma-aminobutyric acid (GABA) ergic inputs into postsynaptic cells become depolarising (or less hyperpolarising).^13^ An important pathway leading to depolarising GABA in divergent preclinical models (nerve injury, diabetes, inflammation) is an increase in intracellular chloride due to a brain-derived neurotrophic factor (BDNF)-induced downregulation of the potassium/chloride cotransporter 2 (KCC2).^9,11,13,34^ In support of a similar mechanistic principle in humans, recordings from lamina I dorsal horn neurons in ex vivo human spinal cord slice preparations also indicate that BDNF can induce a depolarising switch in GABAergic transmission.^11^

This study used HRDD, a noninvasive biomarker of spinal inhibitory function, to investigate the presence of spinal disinhibition in FMS. Hoffman reflex rate-dependent depression is impaired in diabetic rodents that display both behavioural indices of pain and BDNF-dependent depolarising GABA.^33,38^ Importantly, in rodents, HRDD is normalised by interventions (eg, BDNF sequestration) that restore spinal inhibition and alleviate behavioural indices of pain; and it becomes impaired following interventions recapitulating the pathway to depolarising GABA (eg, KCC2 inhibition) that evoke behavioural indices of pain.^30,33^ Although the spinal circuits underlying the deficits in HRDD are not clear,^34^ these findings imply, at least in diabetic rodents, that the same mechanism underlying spinal disinhibition and indices of pain also results in impairment of HRDD. Underpinning the translational potential of these findings, impairment of HRDD is seen in individuals with painful diabetic neuropathy,^38,68^ most evidently in those with prominent mechanical pain hypersensitivity relative to mechanical pain detection.^39^ Unlike for painful diabetic neuropathy, currently, there is no parallel preclinical evidence in FMS to link impaired HRDD with chloride dysregulation in the dorsal horn of the spinal cord. Impairment of HRDD has been demonstrated in individuals with obesity/impaired glucose tolerance,^53^ for which there is an increased incidence in FMS.^12,70^ However, the current data show no correlation between obesity/impaired glucose tolerance and the degree of HRDD impairment. Furthermore, no significant group-level differences in BMI/HbA1c were seen between healthy control participants and individuals with FMS. Therefore, it seems unlikely that the abnormal HRDD in FMS is attributable to a prediabetic state or metabolic syndrome.

A previous investigation has demonstrated an increase in the Hmax:Mmax ratio, which also provides a measure of excitatory-inhibitory balance in the spinal cord,^26^ in individuals with FMS compared with controls.^61^ We did not show any significant alterations in the Hmax:Mmax ratio in individuals with FMS. Both our current findings and the previous study are from a relatively small cohort, potentially contributing to these differing findings. However, the Hmax:Mmax ratio is distinct from HRDD, has different underlying physiological mechanisms,^26^ and has not been associated with spinal disinhibition in clinical or preclinical studies in painful diabetic neuropathy.^33,38^ The nociceptive withdrawal reflex (NWR) has also been used to probe spinal excitability in chronic pain states^37^ with a reduction in threshold being taken as a measure of central hyperexcitability. Whilst in FMS, there is evidence of a reduced NWR threshold, the findings are weak and there is considerable heterogeneity.^64^ Furthermore, unlike impaired HRDD, alterations in the nociceptive withdrawal reflex have not been linked to a specific pain mechanism.

Further evidence of altered processing in the spinal cord comes from functional magnetic resonance imaging studies in individuals with FMS. For example, resting-state functional magnetic resonance imaging of the cervical cord in individuals with FMS has demonstrated a decrease and increase in the amplitude of low-frequency fluctuations in the dorsal and ventral cord, respectively.^41^ The significance of these alterations in the neural processes and whether they relate to spinal disinhibition or impaired HRDD are uncertain. However, they do potentially provide a link between dysfunction in predominant “sensory” and “motor” regions of the spinal cord that could be relevant to abnormalities in HRDD.

Psychophysical evidence of altered central processing—specifically, enhanced temporal summation of pain and inefficient diffuse noxious inhibitory control—has previously been reported in FMS studies.^22,49,55^ Compared with healthy volunteers, we found that temporal summation of pain to pinprick stimuli was enhanced in people with FMS. Temporal summation of pain is widely used as a proxy of processes causing wind-up in the spinal cord,^24^ and our findings could implicate enhanced wind-up in the spinal cord. However, although there is broad agreement that temporal summation is enhanced in FMS across studies,^45^ there is considerable interindividual heterogeneity and, like for HRDD in this study, a lack of correlation with clinical pain.^48^ Between study inconsistencies highlighted in a 2018 meta-analysis revealed that disparate measurement and phenotyping approaches relating to both wind-up and CPM, with the parameters type and site of stimulation, age, sample size, and medication control, may provide important sources of variability.^45^ Indeed, more recent studies using sensitivity-adjusted test stimuli suggest that individuals with fibromyalgia modulate nociceptive input as effectively as healthy volunteers.^56–58^ Whilst we did not use sensitivity-adjusted stimuli in our CPM paradigm, our findings suggest that, in FMS, central mechanisms may evolve over time; both HRDD and CPM were most impaired in the short duration of disease. Similar findings have been reported in patients with pDPN, whereby patients with shorter pain duration demonstrate less efficient CPM and higher temporal summation but with the same level of pain as those with longer duration.^23^ Longitudinal studies are required to investigate the potential time-dependency of central sensitisation mechanisms in FMS, but it is possible that spinal disinhibition and/or alterations in descending modulation could represent an initiating mechanism that subsequently becomes maintained by other processes. If, as these findings suggest, impaired central processing “normalises” with chronicity of pain, it may also explain in part the variance of findings in both research and clinical populations.^22,49,57,58^

Immune mechanisms have emerged as putative aetiopathological factors in FMS and have been linked to peripheral nervous system dysfunction. Passive transfer of IgG from individuals with FMS reproduces the sensory, motor, and pathological abnormalities in mice.^21^ The dominant hypothesis is that autoantibodies bind to satellite glial cells in the dorsal root ganglia (DRG) and sensitise nociceptor fibres.^21^ Whether this leads to secondary changes, including disinhibition, in the spinal cord is not known. Furthermore, as only approximately 50% of individuals display high levels of autoantibody binding to satellite glial cells,^31^ there may be other aetiological mechanisms. We did not measure antibody binding status in this study. Transfer of neutrophils from individuals with FMS to mice also results in reversible somatosensory hypersensitivity, neutrophil infiltration of the DRG, and, interestingly, sensitisation of deep dorsal horn neurons to noxious mechanical and thermal stimuli.^5^ This highlights the capacity of immune-related mechanisms involving the DRG to affect processing in neurons involved in central nociceptor processing and, potentially, segmental spinal reflexes. This is the likely case in painful diabetic neuropathy,^34^ where spinal disinhibition related to chloride dysregulation and impaired HRDD are likely secondary to alterations in peripheral nerves. Among healthy individuals, HRDD shows variation.^68^ An alternative reason for the impaired HRDD in a proportion of individuals with FMS is that spinal inhibitory function, and hence the degree of HRDD, reflects a trait that could prime the development of pain when triggered by an additional insult.

Limitations of this study include its cross-sectional design and relatively small number of participants. In addition, our findings relating to the duration of the disease were a representation of time since diagnosis. As the duration of symptoms before a confirmed FMS diagnosis is on average 5 years and is affected by multiple factors, the precise onset is unlikely to be determined to a single focal point.^6,20^ Furthermore, patients continued to take pain medication during the assessment period, and this was not systematically accounted for in the analysis. Medications would be expected to impact pain ratings, and treatments with antineuropathic pain drugs could potentially differentially alter HRDD.^71^ Larger scale prospective studies, determining longitudinal trends in HRDD, psychophysical parameters, and clinical symptoms and scores over the time course of FMS disease, are required. Larger studies are also needed to determine whether HRDD differs between individuals with FMS with and without small fibre pathology, although impairment of HRDD is not related to the severity of small fibre neuropathy in individuals with diabetic neuropathy.^38,68^ In addition, the response of HRDD to descending modulatory influences, eg, attention, and to antineuropathic therapies that modulate spinal dysfunction/inhibition is required.

In conclusion, we have demonstrated that patients with FMS have impairment of HRDD and therefore evidence of spinal disinhibition. Furthermore, our findings provide evidence that spinal inhibitory function is most impaired in patients with FMS in short-duration disease. Further investigations to expand these findings are needed, as identifying patients with impairment of central pain processing at an early stage may provide opportunities for time-dependent targeted mechanistic-based therapy.

## Disclosures

The authors declare no direct competing interests.

Data sets are available from the corresponding author upon reasonable request, data transfer agreements and associated costs may be required.

## Supplementary Material

SUPPLEMENTARY MATERIAL

## Supplemental digital content

Supplemental digital content associated with this article can be found online at http://links.lww.com/PR9/A279.
