# Supplementary material for: Evidence for spinal disinhibition as a pain-generating mechanism in fibromyalgia syndrome
Source: Pain Rep. 2024 Dec 26;10(1):e1236. doi: 10.1097/PR9.0000000000001236 (PMC11677609; doi:10.1097/PR9.0000000000001236)
Supplement: SUPPLEMENTARY MATERIAL [file painreports-10-e1236-s001.pdf]

Supplementary Table 1. Current pain medication. Number of individuals with FMS and healthy volunteers taking each category/combination of medication. No medication (category green), one medication (category blue), two medication (category peach) and three medications (category orange).

	FMS	HV
No pain medication	10	15
Gabapentinoids	1	0
SNRI	1	0
SSRI	2	2
Tricyclics	1	0
NSAIDS	3	2
Opioids	0	0
Gabapentinoids + opioid	1	0
SNRI + SSRI	1	0
SNRI + opioid	2	0
SSRI + opioid	1	0
SSRI + tricyclic	1	0
Tricyclic + opioid	1	0
NSAID + opioid	3	0
NSAID + SSRI	0	1
Gabapentinoids + opioid + SNRI	2	0
SNRI + opioid + NSAID	1	0
